# Weight loss in people with type 1 diabetes over 12 months: Real‐world data comparing tirzepatide, semaglutide and liraglutide

**DOI:** 10.1111/dom.70172

**Published:** 2025-10-06

**Authors:** Ebaa Al Ozairi, Mohammad Irshad, Jumana Alkandari, Litty Sojan, Dherar Alroudhan, Nourah Alotaibi, Carel W. le Roux

**Affiliations:** ^1^ DAFNE Unit, Clinical Care Research and Clinical Trials Unit Dasman Diabetes Institute Kuwait City Kuwait; ^2^ Amiri Hospital, Ministry of Health Kuwait City Kuwait; ^3^ Diabetes Complications Research Centre University College Dublin Dublin Ireland; ^4^ School of Medicine Ulster University Coleraine UK

**Keywords:** liraglutide, obesity, semaglutide, tirzepatide, type 1 diabetes

## Abstract

**Aims:**

This study aimed to compare the effects of tirzepatide, semaglutide, and liraglutide on body weight and metabolic risk markers over 12 months in people with body mass index ≥27 kg/m^2^ and type 1 diabetes (T1D).

**Methods:**

This real‐world study included 250 people with obesity and T1D (female = 54.8%), treated with either tirzepatide (*n* = 35), semaglutide (*n* = 36), liraglutide (*n* = 97) or usual care (*n* = 82). Secondary outcomes included changes in lipid profile, renal and liver markers, blood pressure, and HbA1c.

**Results:**

All three agents led to significant weight loss. Tirzepatide showed the greatest reduction of weight loss (10.9%; *p* < 0.001), followed by semaglutide (9.9%; *p* < 0.001) and liraglutide (7.1%; *p* < 0.001). Dose‐dependent reductions were observed for tirzepatide and semaglutide. Tirzepatide, semaglutide and liraglutide modestly reduced HbA1c by 0.65% (*p* = 0.004), 0.33% (*p* = 0.034) and 0.23% (*p* = 0.017), respectively. LDL‐cholesterol was reduced by semaglutide (*p* = 0.05) and liraglutide (*p* = 0.02), and liraglutide also lowered the urine albumin‐to‐creatinine ratio (*p* = 0.007). There was no change in body weight and HbA1c in the usual care group. No severe hypoglycaemia or diabetic ketoacidosis (DKA) events were reported in any group.

**Conclusion:**

Tirzepatide, semaglutide, and liraglutide reduced bodyweight and improved in selected metabolic risk markers over 12 months without increasing the risk for hypoglycaemia or DKA. Weight loss appeared less compared with patients without diabetes. Tirzepatide, semaglutide and liraglutide modestly improved glycaemic control in adults with T1D and obesity. These findings support the potential adjunctive role of GLP‐1 receptor agonists in people with obesity and T1D and underscore the need for further validation through randomized controlled trials.

## 1 INTRODUCTION

Insulin therapy is the standard treatment for type 1 diabetes (T1D), but people with T1D can also develop obesity and metabolic risk markers.^1^ The disease of obesity presents a growing challenge in T1D care,^2^ especially with increases in risks of cardiovascular disease, insulin resistance, and diabetic complications.^3^, ^4^

GLP‐1 receptor agonists (GLP‐1 RAs), such as liraglutide, semaglutide and tirzepatide, were developed to treat obesity as they promote satiety, delay gastric emptying and enhance glucose‐dependent insulin secretion.^5^ While these GLP‐1 RAs drugs have become cornerstones in the treatment of obesity,^6^ their use in people with obesity who have T1D has gained attention due to potential benefits on obesity‐related complications, but there has also been an interest in reducing insulin dose requirements and improving glycaemic control.^7^

Liraglutide, a once‐daily GLP‐1 RA, was the first to be studied in patients with obesity and T1D, demonstrating moderate effects on weight and HbA1c.^8^, ^9^ Semaglutide, a once‐weekly formulation, has also shown promising metabolic outcomes in people with obesity and T1D.^10^ Tirzepatide, a combination of GLP‐1 and glucose‐dependent insulinotropic polypeptide receptor agonist, recently approved for obesity, has demonstrated superior efficacy in weight and glucose reduction compared to GLP‐1 RAs alone.^11^, ^12^ However, real‐world data on tirzepatide use in T1D are still emerging.

The present study aimed to compare the real‐world effects of tirzepatide, semaglutide, and liraglutide over 12 months in people with a body mass index (BMI) >27 kg/m^2^ and T1D. We assessed their impact on weight, metabolic risk markers, and glycaemic control.

## 2 METHODS

This real‐world study was conducted using prospectively collected clinical data during routine care at Dasman Diabetes Institute, following ethical approval by the institutional review board. All the participants followed the dose adjustment for normal eating (DAFNE) clinics for routine care. Participants were aged 18 years or older and had received at least 1 year of follow‐up after initiating GLP‐1 receptor agonist therapy. A confirmed diagnosis of T1D was required based on the American Diabetes Association 2022 criteria, including undetectable C‐peptide levels and the presence of diabetes‐related autoantibodies. All participants were treated with insulin, either via continuous subcutaneous insulin infusion (CSII) or multiple daily injections and maintained stable insulin doses for at least 3 months. All individuals had access to blood ketone meters and urine reagent strips for monitoring. Exclusion criteria for both treatment and usual care groups included a BMI below 27 kg/m^2^, pregnancy, use of other oral diabetes or other weight‐loss medications and a history of dementia or psychosis. Additional exclusion criteria were hospitalization or recent episodes of diabetic ketoacidosis (DKA) or severe hypoglycaemia. The participant maintained stable dose GLP‐1 RA for at least 3 months before the follow‐up period ended.

We collected the age, sex, diabetes duration, body weight, height, HbA1c, lipid profile, liver enzymes, and blood pressure from the prospectively completed electronic health records. BMI was calculated as weight in kilograms divided by height in meters squared. Secondary data included complications, adverse events, and treatment discontinuation, defined as a gap of at least 30 days after the expected end date of the last prescription.

### 2.1 Statistical analysis

Statistical analysis was conducted using SPSS Statistics software version 29.0 (IBM Corp, USA) and R software version R 4.2.3. Continuous variables with a normal distribution are reported as mean ± standard deviation (SD), non‐normally distributed variables are presented as median and interquartile range (IQR) and categorical variables are presented as frequency and percentages, where applicable. For the primary outcome (weight change) from baseline to 12‐month follow‐up, analysis was performed using a repeated measures test adjusted for sex and age. Analysis of covariance (ANCOVA) was used to compare weight change between the drugs and usual care group adjusted for age and sex. A propensity score analysis, matched for age, sex, and BMI, was performed to compare weight change for each drug versus the control, and the results are presented in the Supporting Information.

For secondary outcome, within‐group changes from baseline to 12 months follow‐up were evaluated separately using the Wilcoxon signed‐rank test. *p*‐values from the Wilcoxon signed‐rank tests were adjusted using the false discovery rate (FDR) method by Benjamini–Hochberg. Results are reported as mean difference with 95% confidence intervals (CIs) along with both unadjusted and adjusted *p*‐values. A two‐sided adjusted *p*‐value of <0.05 was considered statistically significant.

## 3 RESULTS

A total of 250 people with a BMI ≥27 kg/m^2^ and T1D were using tirzepatide (*n* = 35, 71.1% female), semaglutide (*n* = 36, 72.2% female), liraglutide (*n* = 97, 51.5% female) and usual care (*n* = 82, 41.5% female). Table 1 shows the baseline characteristics that were similar among the four groups, including diabetes duration, body weight, BMI, blood pressure, HbA1c, eGFR, lipid profiles, liver enzymes and daily insulin dose, except for age, sex and urine albumin‐to‐creatinine ratio (ACR).

**TABLE 1:** Baseline characteristics of people with type 1 diabetes according to the medication used.

	Total (*n* = 250)	Usual care (*n* = 82)	Liraglutide (*n* = 97)	Semaglutide (*n* = 36)	Tirzepatide (*n* = 35)	*p*‐value^a^
	Mean (SD) or *n* (%)	Mean (SD) or *n* (%)	Mean (SD) or *n* (%)	Mean (SD) or *n* (%)	Mean (SD) or *n* (%)	
Age (years)	34.4 (9.9)	32.3 (8.9)	34.3 (9.0)	36.6 (11.5)	37.4 (11.5)	0.03
Male	113 (45.2)	48 (58.5)	47 (48.5)	10 (27.8)	8 (22.9)	<0.001
Female	137 (54.8)	34 (41.5)	50 (51.5)	26 (72.2)	27 (77.1)	
Diabetes duration (years)	18.9 (8.8)	18.4 (8.9)	19.1 (8.1)	18.6 (9.2)	19.8 (10.3)	0.87
Body weight (kg)	85.6 (14.7)	84.8 (12.5)	85.9 (14.4)	85.1 (16.3)	86.8 (18.6)	0.91
Height (cm)	164.2 (8.9)	165.3 (9.3)	164.2 (9.0)	162.1 (8.6)	163.8 (7.8)	0.36
BMI (kg/m^2^)	31.6 (4.1)	31.0 (3.3)	31.8 (4.2)	32.2 (4.3)	32.1 (4.9)	0.32
Systolic BP (mmHg)	122.7 (12.9)	122.6 (12.8)	123.4 (12.0)	123.2 (13.0)	120.1 (15.5)	0.63
Diastolic BP (mmHg)	74.5 (9.2)	74.9 (8.6)	74.8 (8.8)	71.8 (11.2)	75.5 (8.9)	0.29
HbA1c (%)	8.1 (1.4)	7.7 (1.4)	8.2 (1.3)	8.1 (1.2)	8.3 (1.5)	0.08
HbA1c (mmol/mol)	64.5 (14.8)	61.1 (15.4)	66.3 (14.1)	65.2 (12.9)	66.8 (16.1)	0.08
ACR (mg/g)	68.3 (253.7)	12.1 (21.1)	131.2 (377.7)	54.3 (176.6)	35.6 (87.3)	0.01
ACR, median (IQR)	7.0 (12.5)	6.0 (6.0)	7.5 (28.0)	8.3 (8.8)	9.0 (14.3)	0.01
ACR (log10)	1.04 (0.62)	0.85 (0.39)	1.17 (0.77)	1.06 (0.59)	1.09 (0.54)	0.01
eGFR (mL/min/1.73 m^2^)	109.6 (20.6)	113.4 (17.6)	108.1 (22.3)	107.5 (22.1)	107.0 (20.7)	0.23
Total cholesterol (mmol/L)	4.6 (1.0)	4.5 (1.0)	4.6 (1.0)	4.7 (0.9)	4.6 (1.2)	0.76
Triglyceride (mmol/L)	0.9 (0.6)	0.8 (0.5)	1.0 (0.7)	0.9 (0.4)	0.9 (0.6)	0.32
LDL‐cholesterol (mmol/L)	2.5 (0.9)	2.4 (0.9)	2.6 (0.9)	2.6 (0.8)	2.4 (1.0)	0.31
HDL‐cholesterol (mmol/L)	1.6 (0.5)	1.7 (0.5)	1.5 (0.4)	1.7 (0.5)	1.8 (0.4)	0.06
ALT (u/L)	29.5 (16.3)	28.5 (14.6)	30.0 (15.9)	31.3 (14.9)	28.7 (22.0)	0.82
AST (u/L)	19.8 (10.1)	21.8 (12.1)	18.1 (6.8)	19.9 (10.7)	19.7 (11.3)	0.11
ALP (u/L)	82.6 (24.8)	78.1 (23.7)	84.2 (25.4)	88.9 (21.6)	82.3 (27.7)	0.15
MDI	198 (79.2)	69 (84.1)	76 (78.4)	26 (72.2)	27 (77.1)	0.49
CSII	52 (20.8)	13 (15.9)	21 (21.6)	10 (27.8)	8 (22.9)	
Insulin dose (unit/day)	58.5 (24.7)	56.5 (21.2)	62.8 (27.2)	56.6 (25.9)	52.9 (23.3)	0.15

Abbreviations: ACR, albumin‐to‐creatinine ratio; ALP; alkaline phosphatase; ALT, alanine transaminase; AST, aspartate transaminase; BMI, body mass index; BP, blood pressure; CSII, continuous subcutaneous insulin infusion; eGFR, estimated glomerular filtration rate; IQR, interquartile range; MDI, multiple daily injections; SD, standard deviation.

^a^ ANOVA test.

After 12 months of treatment, all three GLP‐1 receptor agonists led to significant reductions in body weight, even after adjusting for age and sex. Tirzepatide resulted in the greatest weight loss with a mean reduction of 10.9% (95% CI: 13.2 to 8.6; *p* < 0.001), followed by semaglutide with 9.9% (95% CI: 12.2 to 7.6; *p* < 0.001), and liraglutide with 7.1% (95% CI: 8.5 to 5.7; *p* < 0.001) (Tables 2 and S1). However, no significant change in body weight of the usual care group was observed. Compared with usual care, weight loss remained highest with tirzepatide at 11.2% (95% CI: 15.0 to 7.4; *p* < 0.001), followed by semaglutide at 10.2% (95% CI: 14.0 to 6.5; *p* < 0.001) and liraglutide at 7.4% (95% CI: 10.2 to 4.7; *p* < 0.001) (Tables 3 and S2). Propensity score–matched comparisons of weight change between GLP‐1 receptor agonists and the control group also confirmed that all comparisons remained statistically significant, with the greatest weight loss observed for tirzepatide, followed by semaglutide and liraglutide (Table S3).

**TABLE 2:** Percentage body weight loss after 12 months of treatment.

Drugs	*N*	Weight loss (%)	95% Confidence interval		*p*‐value^a^	Weight loss (%)^b^	95% Confidence interval		*p*‐value^a^
			LB	UB			LB	UB	
Tirzepatide	35	−11.1	−13.4	−8.7	<0.001	−10.9	−13.2	−8.6	<0.001
Semaglutide	36	−10.0	−12.3	−7.8	<0.001	−9.9	−12.2	−7.6	<0.001
Liraglutide	97	−7.0	−8.4	−5.6	<0.001	−7.1	−8.5	−5.7	<0.001
Usual care	82	0.4	−1.1	1.9	0.69	0.3	−1.2	1.8	0.61

Abbreviations: LB, lower bound; UB, upper bound.

^a^ Adjusted: age and sex.

^b^ Repeated measure test.

**TABLE 3:** Percentage of body weight change compared to the usual care.

Drugs	Weight loss (%)	95% Confidence interval		*p*‐value^a^	Weight loss (%)^b^	95% Confidence interval		*p*‐value^a^ ^s^
		LB	UB			LB	UB	
Tirzepatide	−11.4	−15.2	−7.7	<0.001	−11.2	−15.0	−7.4	<0.001
Semaglutide	−10.4	−14.1	−6.7	<0.001	−10.2	−14.0	−6.5	<0.001
Liraglutide	−7.4	−10.2	−4.6	<0.001	−7.4	−10.2	−4.7	<0.001

Abbreviations: LB, lower bound; UB, upper bound.

^a^ ANCOVA test.

^b^ Adjusted: age and sex.

Dose‐stratified analysis revealed a dose‐dependent trend for 2.5–5, 7.5–10 and 12.5–15 mg tirzepatide and 0.25–0.5, 1–1.7 and 2.4 mg semaglutide. For 2.5–5 mg tirzepatide (*n* = 11), weight reductions were 6.5% (95% CI: 11.2 to 1.8; *p* = 0.036), 7.5–10 mg (*n* = 13): 12.4% (95% CI: 16.7 to 8.0; *p* < 0.001), and 12.5–15 mg (*n* = 11): 14.1% (95% CI: 18.9 to 9.3; *p* < 0.001). For semaglutide 0.25–0.5 mg (*n* = 14), the weight loss was 7.9% (95% CI: 12.3 to 3.5; *p* = 0.001), 1–1.7 mg (*n* = 12): 9.5% (95% CI: 14.2 to 4.8; *p* < 0.001) and 2.4 mg (*n* = 10): 13.8% (95% CI: 18.8 to 8.7; *p* < 0.001). For liraglutide 1.8 mg (*n* = 82), weight loss resulted in 6.9% (95% CI: 8.2 to 5.6; p < 0.001) and the 3.0 mg dose (*n* = 15) in 7.7% (95% CI: 10.7 to 4.7; *p* < 0.001) (Table 4).

In addition, tirzepatide was associated with a modest reduction of HbA1c (0.65%, 95% CI: 0.97 to 0.37; *p* = 0.004). Semaglutide was associated with modest reductions of HbA1c (0.33%, 95% CI: 0.54 to 0.13; *p* = 0.034), total cholesterol (0.37 mmol/L, 95% CI: 0.65 to 0.11; *p* = 0.04) and LDL‐ cholesterol (0.29 mmol/L, 95% CI: 0.53 to 0.07; *p* = 0.05). Liraglutide led to a modest reduction of HbA1c (0.23%, 95% CI: 0.36 to 0.11; *p* = 0.017) and a reduction in urine ACR (52.6 mg/g, 95% CI: 94.4 to 19.3; *p* = 0.007), and LDL‐cholesterol (0.25 mmol/L, 95% CI: 0.43 to 0.07; *p* = 0.02). In the usual care group, ALP significantly increased (6.41 u/L, 95% CI: 2.14 to 10.92; *p* = 0.04), AST significantly decreased (2.14 u/L, 95% CI: 4.51 to 0.61; *p* = 0.019), whereas other changes were not sisgnificant (Table 5).

**TABLE 4:** Percentage body weight loss after 12 months of treatment stratified by doses.

Drugs	Dose (mg)	*N*	Weight loss (%)	95% Confidence interval		*p*‐value^a^	Weight loss (%)^b^	95% Confidence interval		*p*‐value^a^
				LB	UB			LB	UB	
Tirzepatide	2.5–5	11	−6.3	−11.0	−1.6	0.049	−6.5	−11.2	−1.8	0.036
	7.5–10	13	−12.0	−16.3	−7.7	<0.001	−12.4	−16.7	−8.0	<0.001
	12.5–15	11	−14.7	−19.3	−10.0	<0.001	−14.1	−18.9	−9.3	<0.001
Semaglutide	0.25–0.5	14	−8.0	−12.8	−3.1	0.002	−7.9	−12.3	−3.5	0.001
	1–1.7	12	−9.4	−14.7	−4.2	0.001	−9.5	−14.2	−4.8	<0.001
	2.4	10	−13.7	−19.4	−8.0	<0.001	−13.8	−18.8	−8.7	<0.001
Liraglutide	1.8	82	−6.9	−8.3	−5.5	<0.001	−6.9	−8.2	−5.6	<0.001
	3	15	−7.8	−11.0	−4.6	<0.001	−7.7	−10.7	−4.7	<0.001

Abbreviations: LB, lower bound; UB, upper bound.

^a^ Repeated measure test.

^b^ Adjusted: age and sex.

**TABLE 5:** Change of clinical indices after 12 months of treatment.

	Mean difference	*p*‐value^a^	*p*‐value^b^
	95% Confidence interval (LB, UB)		
**Tirzepatide**			
HbA1c (%)	−0.65 (−0.97, −0.37)	<0.001	0.004
HbA1c (mmol/mol)	−7.11 (−10.70, −3.94)	<0.001	0.004
ACR (mg/g)	−18.2 (−51.2, 2.6)	0.144	0.230
eGFR (mL/min/1.73m^2^)	0.21 (−3.88, 3.91)	0.773	0.883
Total cholesterol (mmol/L)	−0.35 (−0.65, −0.07)	0.027	0.071
Triglyceride (mmol/L)	−0.14 (−0.31, 0.02)	0.092	0.177
LDL‐cholesterol (mmol/L)	−0.25 (−0.50, −0.01)	0.116	0.192
HDL‐cholesterol (mmol/L)	0.13 (−0.12, 0.53)	0.844	0.883
ALT (u/L)	−4.74 (−13.29, 1.00)	0.601	0.736
AST (u/L)	−3.43 (−7.66, −0.03)	0.046	0.108
ALP (u/L)	−6.40 (−13.20, 0.06)	0.071	0.149
Insulin dose (unit/day)	−11.4 (−16.6, −6.0)	<0.001	0.004
**Semaglutide**			
HbA1c (%)	−0.33 (−0.54, −0.13)	0.009	0.034
HbA1c (mmol/mol)	−3.61 (−5.90, −1.39)	0.009	0.035
ACR (mg/g)	−13.1 (−25.8, −3.5)	0.056	0.122
eGFR (mL/min/1.73m^2^)	1.68 (−0.91, 4.35)	0.220	0.341
Total cholesterol (mmol/L)	−0.37 (−0.65, −0.11)	0.013	0.040
Triglyceride (mmol/L)	−0.10 (−0.22, 0.02)	0.047	0.108
LDL‐cholesterol (mmol/L)	−0.29 (−0.53, −0.07)	0.021	0.050
HDL‐cholesterol (mmol/L)	−0.02 (−0.14, 0.09)	0.614	0.736
ALT (u/L)	−1.67 (−6.89, 3.47)	0.427	0.585
AST (u/L)	0.14 (−3.83, 3.61)	0.797	0.883
ALP (u/L)	−5.17 (−12.33, 1.42)	0.110	0.189
Insulin dose (unit/day)	−8.3 (−13.2, −3.7)	0.004	0.020
**Liraglutide**			
HbA1c (%)	−0.23 (−0.36, −0.11)	0.002	0.017
HbA1c (mmol/mol)	−2.53 (−3.95, −1.16)	0.002	0.017
ACR (mg/g)	−52.6 (−94.4, −19.3)	0.001	0.007
eGFR (mL/min/1.73 m^2^)	−3.16 (−5.84, −0.67)	0.104	0.184
Total cholesterol (mmol/L)	−0.20 (−0.40, 0.00)	0.044	0.108
Triglyceride (mmol/L)	−0.05 (−0.18, 0.07)	0.988	0.988
LDL‐cholesterol (mmol/L)	−0.25 (−0.43, −0.07)	0.004	0.020
HDL‐cholesterol (mmol/L)	0.08 (0.00, 0.17)	0.097	0.179
ALT (u/L)	−0.07 (−3.02, 2.78)	0.979	0.988
AST (u/L)	1.38 (−0.05, 2.84)	0.024	0.067
ALP (u/L)	−3.29 (−7.00, 0.85)	0.004	0.020
Insulin dose (unit/day)	−9.0 (−12.7, −5.3)	<0.001	<0.001
**Usual care**			
HbA1c (%)	0.03 (−0.18, 0.22)	0.481	0.626
HbA1c (mmol/mol)	0.35 (−1.86, 2.40)	0.482	0.626
ACR (mg/g)	0.66 (−2.63, 4.24)	0.685	0.802
eGFR (mL/min/1.73m^2^)	−1.84 (−3.93, 0.23)	0.091	0.177
Total cholesterol (mmol/L)	0.00 (−0.21, 0.21)	0.556	0.702
Triglyceride (mmol/L)	0.09 (−0.08, 0.29)	0.810	0.883
LDL‐cholesterol (mmol/L)	0.01 (−0.15, 0.17)	0.846	0.883
HDL‐cholesterol (mmol/L)	0.04 (−0.03, 0.11)	0.347	0.490
ALT (u/L)	−1.10 (−4.77, 2.30)	0.334	0.486
AST (u/L)	−2.14 (−4.51, 0.61)	0.003	0.019
ALP (u/L)	6.41 (2.14, 10.92)	0.012	0.040
Insulin dose (unit/day)	1.07 (−2.11, 4.13)	0.281	0.421

Abbreviations: LB, lower bound; UB, upper bound.

^a^ Wilcoxon signed‐rank tests.

^b^ False discovery rate (FDR) adjusted *p*‐value.

In the tirzepatide group, daily insulin dose reduction was 11.4 unit/day (95% CI: 16.6 to 6.0; *p* = 0.004); in the liraglutide group, it was 9.0 unit/day (95% CI: 12.7 to 5.3; *p* < 0.001) and in the semaglutide group, 8.3 unit/day (95% CI: 13.2 to 3.7; *p* = 0.02). No adverse effects, such as severe hypoglycaemia or DKA, were reported during the study period in any of the three groups.

## 4 DISCUSSION

This study compared the effectiveness of three modern obesity medications, tirzepatide, semaglutide and liraglutide in people with a BMI ≥27 kg/m^2^ and T1D. The results demonstrate that all three drugs dose‐dependently reduced body weight after 12 months of treatment, with tirzepatide showing the most substantial effect, followed by semaglutide and liraglutide. These findings align with previous studies in people with obesity with or without type 2 diabetes (T2D), where tirzepatide has superior weight loss outcomes compared to semaglutide.^11^, ^13^ The dose‐dependent weight reductions are consistent with earlier trials, including the SURPASS program and STEP trials.^14^, ^15^ This suggests that individualized titration can enhance therapeutic outcomes and minimize side effects, similar to trends observed in other studies in T1D.^16^ The magnitude of weight loss appears to be more similar than the magnitude of weight loss in people with T2D and obesity compared to people without diabetes and obesity.^15^, ^17^, ^18^, ^19^

Tirzepatide, semaglutide and liraglutide also led to modest improvements in HbA1c. Our comparison was likely underpowered because of the small effect size and large variability of HbA1c changes. Our findings align with recent observational data showing greater weight loss and modest glycaemic improvements with tirzepatide in real‐world T1D populations. In a study, weight loss was greater in the tirzepatide group (21.4%) compared to the semaglutide group (9.1%), while modest reductions in HbA1c were observed in both groups (−0.68% for tirzepatide and −0.54% for semaglutide) over 12 months.^16^ Another study reported a 21.4% reduction in body weight and a 0.68% decrease in HbA1c over the 12‐month period.^20^ While Akturk et al. found an 8% reduction in body weight and a 0.59% decrease in HbA1c over 8 months in people with T1D.^21^ These effects are likely due to a reduction in insulin resistance making it possible to use lower doses of insulin but also making it easier to titrate the insulin dose to optimize glycaemic control.^22^ Taken together, the weight loss effects appear robust, but these medications should not be considered if the primary objective is glycaemic control.

The semaglutide‐induced reductions in total cholesterol and LDL‐cholesterol were consistent with improved cardiometabolic risk in individuals with obesity and T1D. These findings align with previous research reporting statistically significant but clinically minor reductions in LDL‐cholesterol and triglycerides, along with improvements in HbA1c and body weight in people with obesity and T1D treated with semaglutide.^23^

Liraglutide also led to reductions in body weight, urine ACR, eGFR and LDL cholesterol. Our data were consistent with findings from the ADJUNCT ONE and ADJUNCT TWO trials that used liraglutide as an adjunct to insulin in people with obesity and T1D.^24^, ^25^ While most cardiovascular data are available for people with obesity and T2D, our findings are also consistent with those of the LEADER trial; the latter demonstrated reduced cardiovascular risk with liraglutide compared to placebo.^26^, ^27^

Importantly, no adverse events such as DKA or severe hypoglycaemia were observed during the 12‐month treatment period, indicating a favorable safety profile in this cohort. These results are consistent with previous studies demonstrating the tolerability of GLP‐1 RAs in people with obesity and T1D when used alongside insulin therapy.^20^

The limitations of this study include the prospective and observational nature, limiting causal inference. The sample sizes were small and unequal. Medication adherence and insulin dosing data were not controlled between the groups as this was a real‐world study. Additionally, patient selection bias cannot be excluded as this was not a randomized controlled trial. In the adverse events data, we only recorded severe hypoglycaemia and DKA and did not capture other adverse events such as gastrointestinal problems, nausea, and vomiting, which limit our findings. We were surprised that no severe hypoglycemia or DKA was reported and verify that our data were correct. Additionally, we did not measure the participants' body composition, so the observed total body weight loss may also include fat‐free mass loss, highlighting the need for further randomized studies. Future larger datasets should have an additional analysis comparing males and females.

In conclusion, modern obesity medications significantly reduce weight in people with obesity and T1D. Tirzepatide showed the most pronounced weight loss effects, with only modest improvement in glycaemic control compared to semaglutide and liraglutide. The amount of weight loss over 12 months appears lower than would be expected from patients without diabetes. These results support the integration of obesity medications as adjunctive therapy in people with the disease of obesity who also have type 1 diabetes. Confirmation in prospective randomized controlled trials will provide scientific evidence to allow large‐scale treatment of people with obesity and T1D.

## AUTHOR CONTRIBUTIONS

*Conceptualization and design*: EA, MI and ClR. *Conduct/data collection*: MI, LS, JK, DA and NA. *Analysis and interpretation of the data*: EA, MI and ClR. *Drafting the manuscript and critical review*: MI, ClR and EA. All authors accessed and verified the underlying manuscript data and approved the final version of the manuscript.

## FUNDING INFORMATION

The manpower has been funded by the Kuwait Foundation of Advancement of Science (KFAS) and the Ministry of Health, Kuwait. The funding agency did not influence the study design, data analysis, interpretation, or report preparation.

## CONFLICT OF INTEREST STATEMENT

Carel W. le Roux has received personal fees from Boehringer Ingelheim, Eli Lilly, GI Dynamics, Gila Pharmaceuticals, Herbalife, Johnson & Johnson, Keyron, Novo Nordisk, and Zealand Pharma outside the submitted work. Alexander D Miras has received research funding from the Medical Research Council, National Institute of Health Research, Jon Moulton Charitable Foundation, Fractyl, Gila, Randox and Novo Nordisk. ADM is a shareholder in the Beyond BMI clinic, which provides clinical obesity care. Other authors declared that they have no competing interest. All authors meet criteria for authorship as recommended by the International Committee of Medical Journal Editors (ICMJE) and did not receive payment related to the development of this manuscript.

## ETHICS STATEMENT

The study was approved by the Dasman Diabetes Institute Ethical Review Committee, Kuwait, and followed the guidelines set out in the Declaration of Helsinki.

## Supporting information


**Table S1.** Body weight loss (kg) after 12 months of treatment.
**Table S2.** Body weight change compared to the usual care.
**Table S3.** Propensity score–matched comparisons of weight change (Drug vs. Control).

## Data Availability

To ensure independent interpretation of clinical study results and enable authors to fulfill their role and obligations under the ICMJE criteria, Dasman Diabetes Institute grants all external authors access to relevant clinical study data. In adherence to the Dasman Diabetes Institute Policy on Transparency and Publication of Clinical Study Data, scientific and medical researchers can request access to clinical study data after the publication of the primary manuscript and secondary analyses in peer‐reviewed journals.
