# Supplementary material for: Weight loss in people with type 1 diabetes over 12 months: Real‐world data comparing tirzepatide, semaglutide and liraglutide
Source: Diabetes Obes Metab. 2025 Oct 6;28(1):166–73. doi: 10.1111/dom.70172 (PMC12673458; doi:10.1111/dom.70172)
Supplement: Supplementary file 1 — Table S1. Body weight loss (kg) after 12 months of treatment. Table S2. Body weight change compared to the usual care. Table S3. Propensity score–matched comparisons of weight change (Drug vs. Control). [file DOM-28-166-s001.docx]

**Supporting Information**

Table S1: Body weight loss (Kg) after 12 months of treatment.

| Drugs | Weight loss (Kg) | 95% Confidence Interval | | P-value# | Weight loss (Kg)* | 95% Confidence Interval | | P-value# |
| --- | --- | --- | --- | --- | --- | --- | --- | --- |
|  |  | LB | UB |  |  | LB | UB |  |
| Tirzepatide | -10.1 | -12.3 | -8.0 | <0.001 | -10.2 | -12.4 | -8.1 | <0.001 |
| Semaglutide | -8.1 | -10.2 | -6.0 | <0.001 | -8.2 | -10.3 | -6.1 | <0.001 |
| Liraglutide | -6.0 | -7.3 | -4.8 | <0.001 | -6.0 | -7.3 | -4.8 | <0.001 |
| Control | 0.3 | -1.1 | 1.7 | 0.69 | 0.4 | -1.0 | 1.8 | 0.61 |

*Adjusted: age and sex

# Repeated measure test

LB, lower bound; UB, upper bound

Table S2: Body weight change compared to the usual care.

| Drugs | Weight loss (Kg)† | 95% Confidence Interval | | P-value* | Weight loss (Kg)‡ | 95% Confidence Interval | | P-value* |
| --- | --- | --- | --- | --- | --- | --- | --- | --- |
|  |  | LB | UB |  |  | LB | UB |  |
| Tirzepatide | -10.3 | -13.6 | -6.9 | <0.001 | -9.7 | -13.1 | -6.2 | <0.001 |
| Semaglutide | -8.4 | -11.7 | -5.1 | <0.001 | -7.9 | -11.3 | -4.6 | <0.001 |
| Liraglutide | -6.2 | -8.7 | -3.7 | <0.001 | -6.1 | -8.5 | -3.6 | <0.001 |

*ANCOVA test

†Covariate: Baseline weight

‡ Covariate: Baseline weight, age and sex

LB, lower bound; UB, upper bound

Table S3: Propensity score–matched comparisons of weight change (Drug vs. Control)

| Comparison | Matched N (pairs)* | Weight loss (Kg) | 95% Confidence Interval (LB, UB) | p-value** | p-value# |
| --- | --- | --- | --- | --- | --- |
| Tirzepatide vs Control | 28 | -9.8 | (-13.6, -6.1) | <0.001 | <0.001 |
| Semaglutide vs Control | 19 | -8.8 | (-12.6, -5.0) | <0.001 | <0.001 |
| Liraglutide vs Control | 46 | -5.9 | (-8.43, -3.4) | <0.001 | <0.001 |

*Each drug was matched 1:1 with control using propensity-score estimates using baseline covariates: sex, age, weight, HbA1c, eGFR, lipid profile, liver enzymes, blood pressure, insulin regimen, and insulin dose.

**Mann-Witney U test

#False discovery rate (FDR) adjusted p-value.

LB, lower bound; UB, upper bound
